# Customizing the Types of Technologies Used by Patients With Type 1 Diabetes Mellitus for Diabetes Treatment: Case Series on Patient Experience

**DOI:** 10.2196/11527

**Published:** 2019-07-09

**Authors:** Anna Holubová, Martina Vlasáková, Jan Mužík, Jan Brož

**Affiliations:** 1 Spin-off Company and Research Results Commercialization Center First Faculty of Medicine Charles University Prague Czech Republic; 2 Department of Information and Communication Technologies in Medicine Faculty of Biomedical Engineering Czech Technical University in Prague Kladno Czech Republic; 3 Department of Internal Medicine Second Faculty of Medicine Charles University Prague Czech Republic

**Keywords:** type 1 diabetes mellitus, technology, self-management, wearable electronic devices, education, telemedicine

## Abstract

**Background:**

Despite the fact there are many wearable and mobile medical devices that enable patients to better self-manage their diabetes, not many patients are aware of all the options they have. In addition, there are those who are not fully satisfied with the devices they use, and those who often do not use them effectively.

**Objective:**

The study aimed to propose possible changes to the combination of devices used by 6 specific patients for diabetes self-management. We assessed the suitability of selected technical devices for diabetes control.

**Methods:**

Data of 6 patients (3 men and 3 women) with type 1 diabetes mellitus, who had been using the Diani telemedicine system for at least 3 months, were analyzed. The suitability of selected technical devices for diabetes control was ascertained using the data obtained via the Diani telemedicine system, as well as the patients’ subjective feelings and statements, their everyday life habits, and self-management of diabetes. Informed consent was signed and obtained from each of the patients included.

**Results:**

Each of the presented case studies describes how a given patient handled the system and its specific components based on his or her lifestyle, level of education, habits related to diabetes management, personality type, and other factors. At the conclusion of each case study, the best composition of devices for patients with similar personal descriptions was suggested.

**Conclusions:**

We believe this study can provide relevant guidance on how to help particular patients choose the technology that is best suited for their needs, based on the specific patient information we are able to obtain from them. Furthermore, clinicians or educators should be aware of available technologies a given patient can choose from. In addition, there is a substantial need for proper patient education in order for them to effectively use devices for diabetes self-management.

## Introduction

### Background

Diabetes mellitus is a metabolic disease associated with the development of chronic complications. Slowing down or stopping the progression of these complications is associated with sufficient diabetes control, that is, maintaining blood glucose values within the recommended targets.

Despite the fact that various types of mobile and Web apps, wearable medical or fitness devices, and telemedicine solutions are being developed to help achieve the goals of diabetes control [1-5], not many patients are aware of all the options they have or are not fully satisfied with the devices they use and, besides that, they often do not use them effectively [6-10].

This is because of the lack of (1) time clinicians can spend with patients during consultation and (2) the information about all the technological possibilities [11-13]. Therefore, patients are often provided with a device without an option to make a choice for themselves or detailed consultation about both its proper use and the option that would fit them the most.

However, it is very important to learn from how different types of patients use a given technology to understand what does truly help them in their self-management, what increases their adherence, what are their preferences, or otherwise, what are the drawbacks and limitations that keep them from using the technology itself. A deeper understanding of a patient’s needs and abilities with respect to the self-management can help us to both tailor a given device for a particular group of patients and assemble the set and types of devices that comply with a patient’s needs the most.

### Objectives

This study aims to analyze the way of using different combinations of technologies for diabetes self-management in 6 specific patients with type 1 diabetes mellitus (T1DM) and, based on such an analysis, to propose possible changes in such combinations that would improve patients’ overall satisfaction and adherence to its use.

## Methods

### Aim

The study aimed to analyze the suitability of selected technical devices for diabetes control based on data obtained via the Diani telemedicine system, patients’ subjective feelings and statements, their everyday life habits, and self-management of diabetes.

### Inclusion Criteria

The inclusion criteria were adults with T1DM for a duration of at least 1 year who had used the Diani telemedicine system for at least 3 months; completed a minimum of 2 consultations with the doctor and the technology educator, that is, before and 3 months after the system use; and shared information about their daily regimen, self-management, and experiences with diabetes-related technologies so far during the consultations.

### Study Sample

The study included 6 patients (3 men and 3 women with average age of 43 [SD 23] years) with T1DM for a duration of at least 1 year, using specific combination of devices and having an experience with the Diani telemedicine system for 3 months (n=3), 6 months (n=1), or 4 years (n=2) in different times for the last 4 years. All the participants were of Czech nationality.

### Collected Data

The data obtained from the Diani telemedicine system included blood glucose values transferred from a connected glucometer and a continuous glucose monitor (CGM), step counts collected via an activity tracker in 1-min intervals, and carbohydrate intake (in grams) and insulin injections manually registered in a connected mobile app.

### Telemedicine System

The Diani telemedicine system is being developed under the university project of the First Faculty of Medicine (Charles University in Prague) and the Faculty of Biomedical Engineering (Czech Technical University in Prague). The system consists of wearable technologies, namely an activity tracker, a blood glucose meter, a diabetes diary mobile app, a smartwatch, and a Web app into which all the data from the wearables are synchronized automatically. The blood glucose meter transfers measured glucose values into the diary mobile app via Bluetooth. The data from the activity tracker are synchronized via smartphone as well. Using the smartwatch, the user can not only track his/her last data registrations about blood glucose, carbohydrate intake, insulin dose, and physical activity (PA) but can also use the watch to make these registrations, which are then transferred to the mobile app via Bluetooth [14]. Occasionally, some of the patients may wear the CGM that enables them to transfer data to the Diani Web app (see Multimedia Appendix 1) either automatically (using the xDrip device, The Nightscout Project) or manually (uploading the raw data file through the Diani Web app) [15-18].

### Patient Instructions

Before using the system, each of the patients was properly instructed by a technology educator on how to operate each of the system components, and they were free to decide which data and how frequently they want to enter the data into the diary. The patients could choose not to use any of the devices if they felt uncomfortable using them.

Before starting to use the system and during the monitoring phase, the patients took part in interviews with a doctor and the technology educator about their daily regimen, technology capabilities, life preferences, and similar topics. The technology educator was also tracking patterns of handling the devices while educating the patients on how to use them. During the phase in which the patients were using the system in their daily life, the educator was also monitoring their behavior of entering and collecting the data via the Diani Web app. These types of interventions represented the most important approaches to get relevant feedback about the usability of the system.

The study involving human participants was conducted in accordance with the Helsinki Declaration and has been approved by the ethics committee of the Motol University Hospital in Prague. Informed consent was signed and obtained from each of the patients included.

### Outcomes

The outcomes included glycated hemoglobin (HbA_1c_) obtained from the patient’s records of his/her caregiver, the average number of data registrations, and the frequency of hypoglycemia.

## Results

### Data Analysis

A total of 6 patients who met the criteria for the intervention were analyzed, based upon the collected data and individual face-to-face consultation.

General description of each patient can be seen in Table 1. The results from data evaluation are shown in Tables 2 and 3.

### Case Series

Each of the following 6 case series describes how a given patient was handling the system and its particular components based on his/her lifestyle, level of education, manners in diabetes management, personality type, and other factors. At the end of each case study, we then propose the best composition of devices for patients with similar personal needs.

#### Case Study 1

##### Patient Information

A 45-year-old woman was diagnosed with T1DM in 2013, and since then, she has been on multiple daily injection (MDI) insulin therapy. Besides her diabetes, she is not suffering from any other diseases or diabetes complications.

##### Daily Regimen and Self-Management

This patient performs PA such as cycling, workouts in a gym, and others for 2 to 4 hours daily. Being a teacher with a stable daily schedule, she can include regular PA into her daily activities.

To keep her blood glucose within the target range and maintain a slim figure, she maintains a lower carbohydrate diet (approximately 100 g/day) and healthy food intake, besides the PA performance. She is able to maintain her blood glucose mostly within the target range, with a very rare occurrence of clinically important hypoglycemia [19] (see Table 3). The only problem she has is that of higher blood glucose levels at night probably caused by the later effect of fatty cheese and nuts she is used to eating later in the evening.

**Table 1:** Demographic and baseline characteristics.

Patient #	Gender	Age (years)	Type 1 diabetes mellitus duration (years)	Current therapy regimen	CGM^a^ use experience for the last 1 year (patients’ subjective evaluation)	Duration of Diani system use
1	Female	45	5	MDI^b^	Few times a year	3 months
2	Female	29	13	CSII^c^	Full time	6 months
3	Female	24	19	CSII	Full time	4 years
4	Male	27	26	CSII	Few times a year	4 years
5	Male	45	4	MDI	None	3 months
6	Male	87	36	MDI	None	3 months

^a^CGM: continuous glucose monitor.

^b^MDI: multiple daily injection.

^c^CSII: continuous subcutaneous insulin infusion.

**Table 2:** Data obtained from 3-month period of using the Diani system.

Patient #	Number of days with continuous glucose monitor	Average number of self-measured blood glucose per day^a^	Average number of carbohydrate registrations per day^b^	Average number of insulin registrations per day^b^	Average number of physical activity registrations per day^b^	Average number of step counts per day^a^
1	18	4.4	5.5	4.1	1.47	14,367
2	72	5.9	3.6	3.8	0.34	9309
3	30	6.6	1.4	1.6	0.18	10,888
4	6	4.3	2.0	5.1	0.60	10,350
5	0	2.5	0	2.9	0.03	4299
6	0	3.2	0.6	0.4	0.05	—^c^

^a^Measured values automatically transferred to a connected mobile app.

^b^Data manually registered to the diabetes diary mobile app.

^c^Missing data.

**Table 3:** Glycated hemoglobin (HbA_1c_) values and frequency of hypoglycemia.

Patient #	HbA_1c_ before using the system (mmol/mol)	HbA_1c_ after 3 months’ experience (mmol/mol)	HbA_1c_ after 6 months’ experience (mmol/mol)	HbA_1c_ after 4 years’ experience (mmol/mol)	Average number of self-measured hypoglycemia <3.9 mmol/L per week during the first 3-month period	Average number of self-measured hypoglycemia <3.0 mmol/L per week during the first 3-month period
1	51	48	—^a^	—	2.6	0.08
2	65	54	47	—	4.8	0.38
3	66	63	69	47	5.7	1.03
4	78	62	69	54	4.4	1.91
5	63	54	—	—	0.6	0.07
6	67	71	—	—	1.3	0.78

^a^Not applicable.

##### Patient’s Attitude to Technology

**Figure 1:**
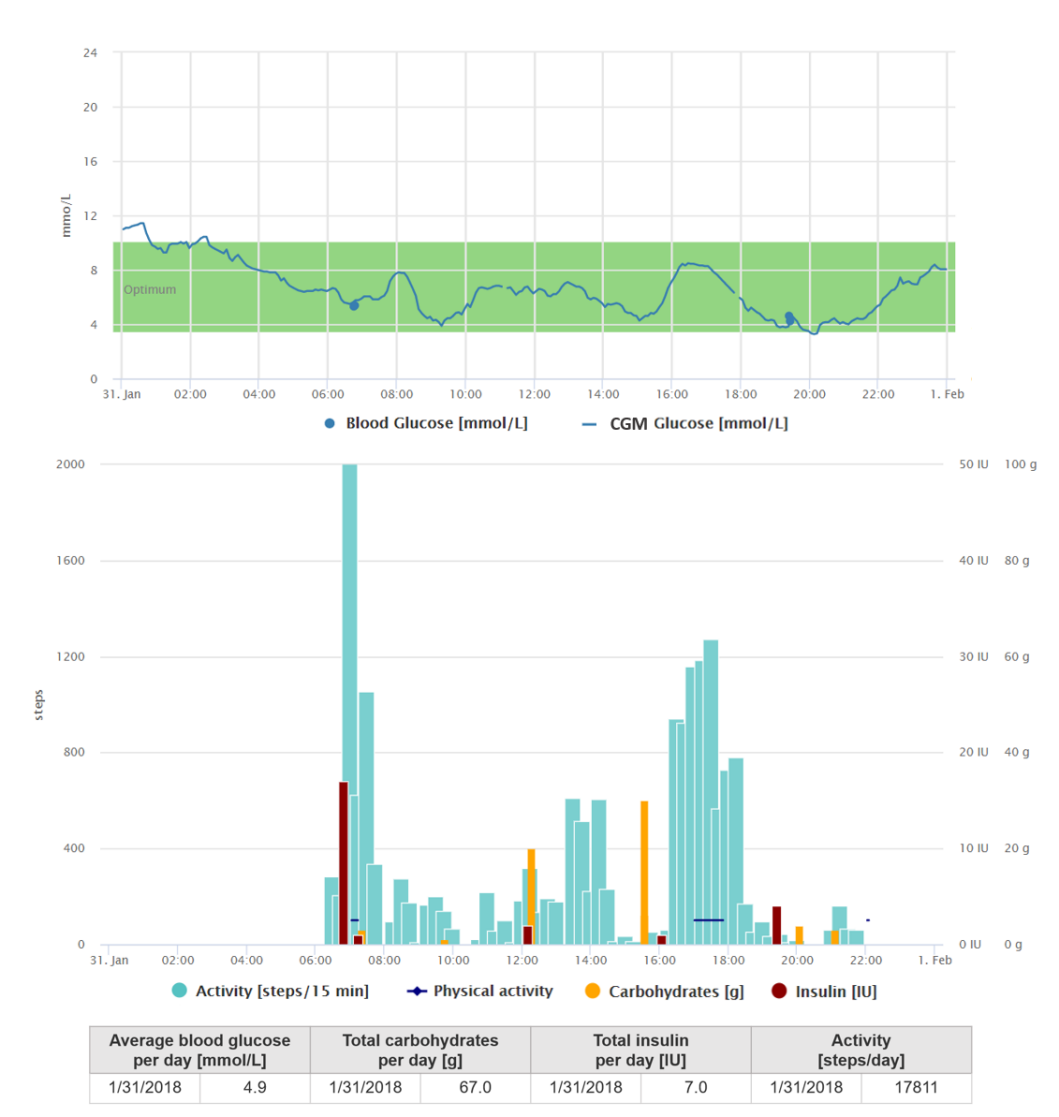
Graphical interpretation of 1-day data registrations of the patient from case study 1, visualized by the Diani Web app. In the picture, we can observe patterns, such as the low-carbohydrate intake, high intensity of physical activity, and higher glucose values during the night. CGM: continuous glucose monitoring.

Since the last year, she has been using 3 sensors for CGM monitoring, but besides that she has relied on self-measured blood glucose (SMBG) alone. Considering her higher educational level and age, she is familiar with using a smartphone and wearable technologies and has no problem to intuitively and quickly learn how to operate a new mobile app or a device for self-management. She has no problems wearing an activity tracker on a full-time basis (during the day and at night). She is also conscientious with respect to registering data into the diabetes diary app (see Table 2) and regularly reviews her data via both mobile and Web apps. She is used to discussing her diabetes difficulties with her clinician, using collected data. Many of these features can be seen on a 1-day graph (Figure 1) representing her regular day.

However, what troubles her are devices that are uncomfortable to wear when performing PA (for this reason, she was not willing to use the smartwatch) or treatment-related devices that are visible to other people. She tends to conceal her disease from the people around her very carefully and makes any treatment actions as discreetly as possible. Therefore, she fully refuses to use an insulin pump and prefers to inject the insulin with a pen in private areas (despite the usefulness of flexible dosing during PA that the insulin pump could enable her). She is willing to wear the CGM sensor only in places that are not visible behind the clothes from the outside.

The biggest benefit she gained from the telemedicine system after she started to use it was that she realized her blood glucose was affected by certain foods and drinks which she had not been covering with insulin dose. Another new information was a postmeal spike after a larger portion of carbohydrate intake, which was connected to a too short time span between the insulin dose and carbohydrate intake. In the long run, she can benefit from controlling her stable total daily dose of carbohydrates and total daily step count, in addition to the blood glucose measurements. By tracking her daily step counts and intensity of PA for a specific sport, she was able to compare her activity level with other friends. The knowledge that there had been nobody who would make a better performance made her feel even more motivated to continue in such regimen. Wearing a CGM helped her mainly to control her blood glucose during PA and discover the postmeal spikes. The receiver clipped on her pants did not represent any obstacle for her.

##### Suggestions for the Optimal Combination of Devices

The ideal tailored system for this type of patient could be a combination of devices that would transfer all the data into a mobile app or display the values on a screen of devices, which would not represent a stigma for them or be visible to the people around them. A thin wristband sensor for tracking activity and a sensor for glucose control would certainly be a good choice. No frequent notifications or alarms from the data analysis would probably be necessary, as this patient is able to review the data regularly. The alarms coming from the CGM system would need to be in vibration mode only to comply with the discretion requirements. Another option could be switching for a flash glucose monitoring, which would be a secret form of data capturing in case the patient would not mind not receiving alarms at night and could wear it on alternative places of the body. The implantable sensor could also work if being implanted in places, which the patient could cover with summer clothes.

Regarding the insulin therapy, insulin pen treatment still seems to be an option complying with the patient’s requirements because of her rejection of wearing an insulin pump. However, improvement in this area could be at least a pen enabling to transfer data to a mobile app via Bluetooth. As the patient is capable of operating digital technologies, we could also try to shift her to a patch pump therapy, if that would be acceptable for her to wear, rather than the traditional pumps with tubing. She could then effectively reduce nighttime highs using a squared bolus or temporary basal rate settings for the high-fat snacks intake she has a difficulty to control.

A personal account for a Web app connected to a clinician’s account for automatic data synchronization would be a matter of course.

#### Case Study 2

##### Patient Information

A 29-year-old woman has T1DM since 2005, and she has been on continuous subcutaneous insulin infusion (CSII) regimen since 2013. Besides her diabetes, she is not suffering from other diseases or diabetes complications. She is preparing for pregnancy.

##### Daily Regimen and Self-Management

This patient is specific in her motivation to improve her HbA_1c_ because she is planning for pregnancy. Being a high school teacher, her daily program is stable and regular. Some unexpected changes in her schedule or emergencies can, however, occur occasionally. These events mostly influence her ability to eat regularly and on time. She is used to maintaining a healthy food intake, but to a certain extent, she has to adapt to the menus at the school canteen during lunchtimes.

She likes walking a lot and she goes for a long walk every day, if the time allows her to do so. She is also educated in flexible dosing of insulin and tries to make changes in her insulin dosing herself based upon the collected data. From time to time, she struggles with nighttime hypoglycemia, mostly induced by evening walks.

##### Patient’s Attitude to Technology

Regarding her technical abilities, she can learn how to use any device easily if she gets sufficient instructions and can turn to technical support or a more advanced person when she gets into trouble, for example, with Bluetooth connection or unpaired devices. She is a smartphone user, so any kind of mobile apps do not pose any obstacle for her to install and use.

She is not concerned about how many devices she has to wear or whether they are visible to the people around her. The accuracy of blood glucose measuring devices is more important to her than the design and size of the technology.

She has been on the CGM system full time since November 2017. At the same time, she started to use all the other equipment within the Diani system. She also took advantage of sharing CGM data with her T1DM friend as another motivation to achieve better blood glucose results while being observed by another person.

By wearing the activity tracker, she has been motivated to walk more and compete with friends who track their daily steps as well.

Regarding her manual registrations, she has entered her insulin doses and carbohydrate information into the diary almost daily, but some lack of data within a day or several days with a pause of manual registrations occurs. She reviewed her historical data more frequently at the beginning to identify problems that caused her blood glucose fluctuations. Once she stabilized her glycemia, she started reviewing the data only a few days before the visit to her diabetologist. She always prepares for the visit and downloads the data for the doctor.

**Figure 2:**
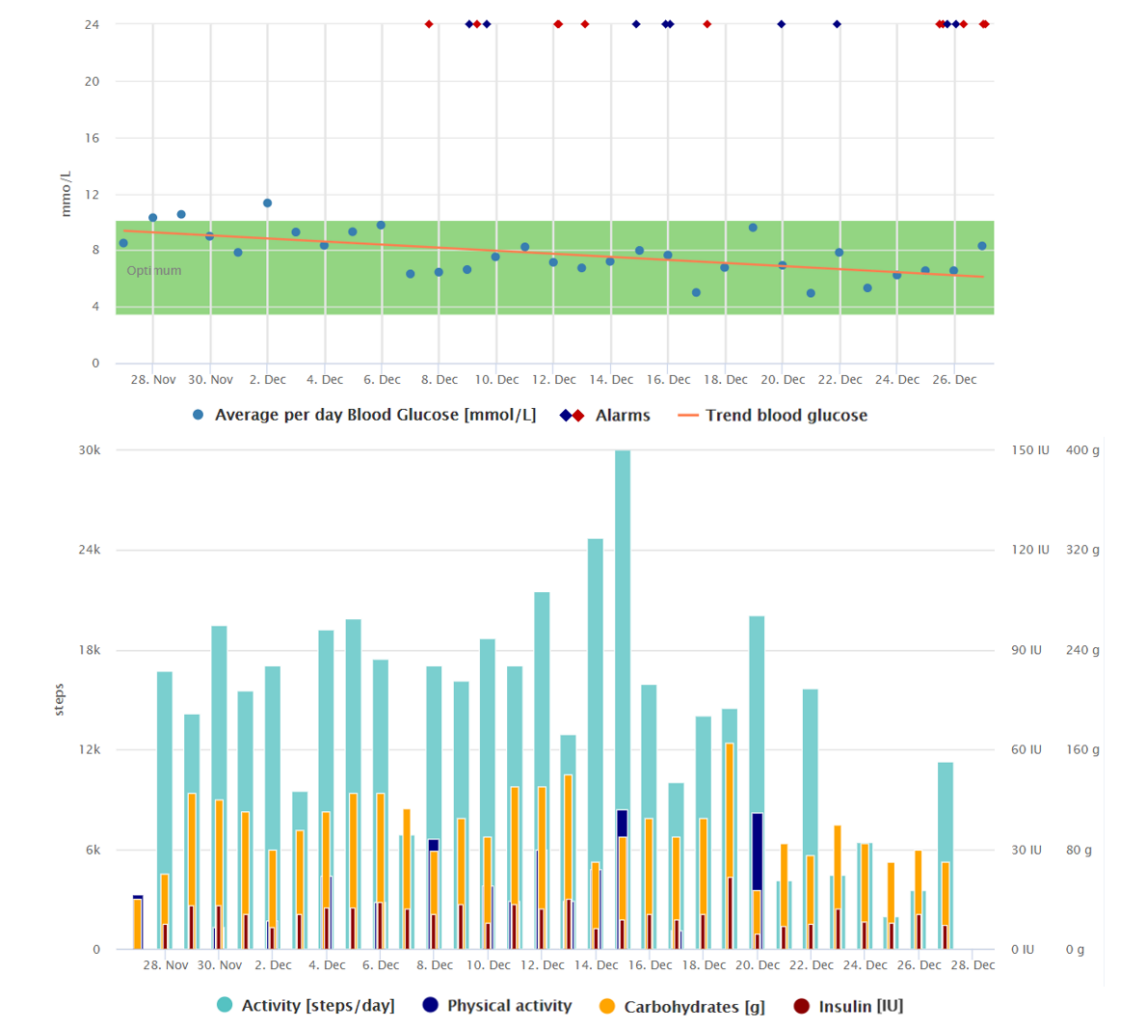
Graphical interpretation of 1-month data registrations of the patient from the case study 2, visualized by the Diani Web app. In the picture, we can observe everyday data registrations, high daily step counts, and decreasing trend of average daily blood glucose.

**Figure 3:**
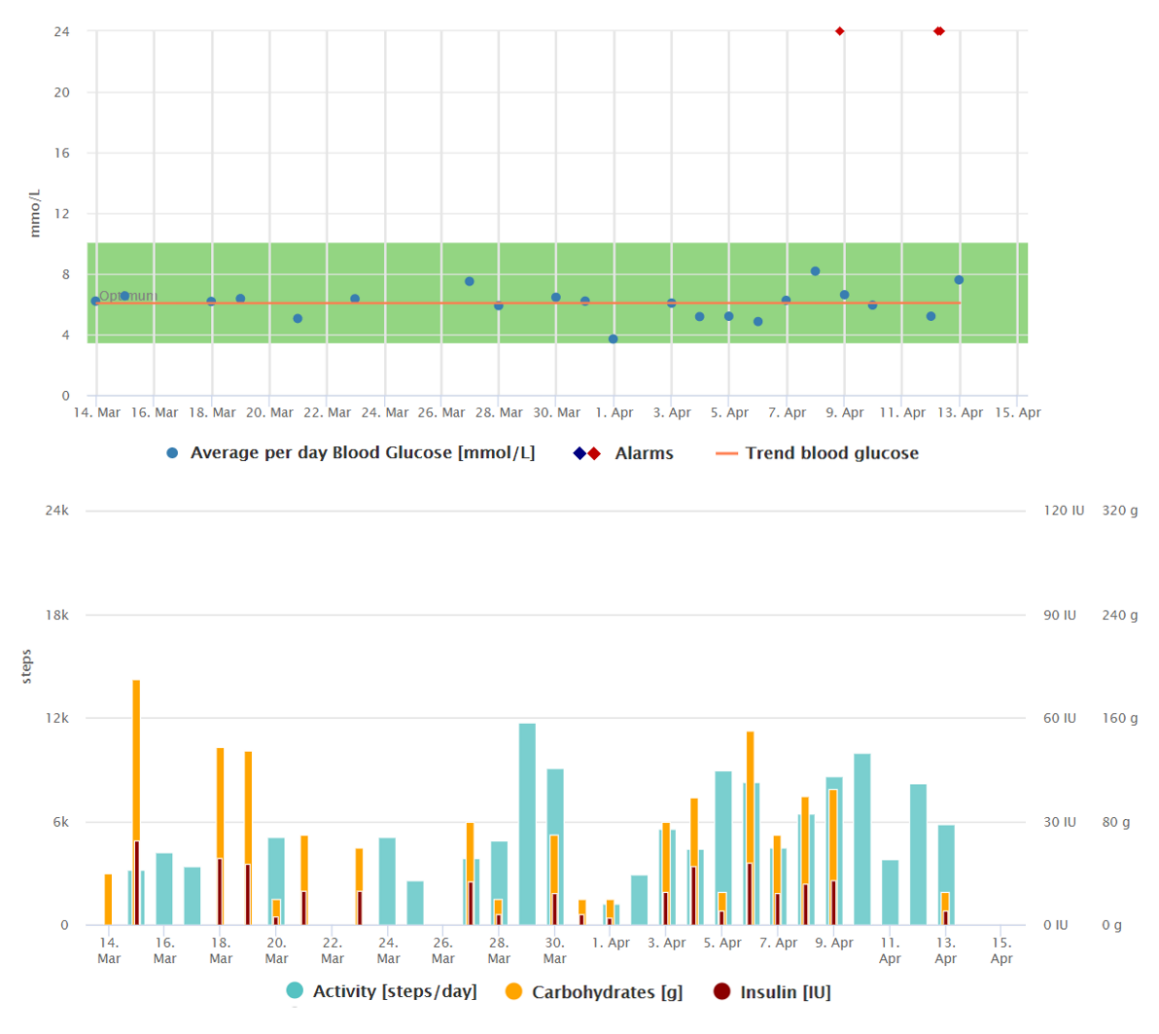
Graphical interpretation of 1-month data registrations of the patient from case study 2, visualized by the Diani Web app.

The trend indicating her blood glucose improvement and the way she collects the data can be seen in Figures 2 and 3. Compared with Figure 2, we can see that the regular data registrations are reduced after the average blood glucose has stabilized.

##### Suggestions for the Optimal Combination of Devices

Ideal tailored system for this type of patient would certainly be an insulin pump in combination with a CGM system that would enable her to transfer data to mobile and Web apps. As we know she experiences nighttime hypoglycemia, the flash glucose monitoring would not be beneficial for her, despite the ability to use it in combination with a smartphone. Knowing her strong focus on device accuracy, the most accurate CGM device enabled with alarm and data transfer to a mobile app might be a good option for her blood glucose monitoring. Considering her motivation while sharing data, a device having such a function included would represent another preference point. Regarding the type of a pump, she is not an exacting user and uses its basic settings and functions (normal boluses, temporal basal reduction, and 1 basal profile). The most convenient option would ideally be a pump that can receive data from the CGM device, in addition to the CGM data synchronization with a smartphone. This would ensure both the ability of data sharing and assurance of data transfer to the pump in case the smartphone has weak signal or its battery level is low. If no such device is available to her, she could choose a pump that is at least comfortable to wear, easy to use, enables to export data for a doctor, and has good technical support.

#### Case Study 3

##### Patient Information

A 24-year-old woman was diagnosed with T1DM in 1999, and she started with CSII therapy in 2002. She suffers from knee pain because of patellofemoral dysplasia, which she was diagnosed with in 2017. Besides that, she is not suffering from other diseases or diabetes complications.

##### Daily Regimen and Self-Management

This patient is a university student and, in addition, has a part-time job. Therefore, her daily program is changing frequently, and she is often busy with work until late evening.

As she struggles with a higher insulin resistance and complications with her knees, she tries to reduce her weight by decreasing her total daily carbohydrate intake and incorporating different types of PAs in her daily program. From time to time, she makes an exception in her regular dietary plan and takes a high-carbohydrate food because she is able to anticipate the majority of her postmeal spikes using her knowledge from the CGM data. She is very well educated and able to keep her blood glucose in a tight target range, mostly thanks to her regular attendance of educational courses and CGM monitoring throughout the whole year.

##### Patient’s Attitude to Technology

This patient is very interested in new wearable technology not only for diabetes treatment. Wearing any kind of mobile technology, even visible medical devices, is not perceived as a stigma by her.

**Figure 4:**
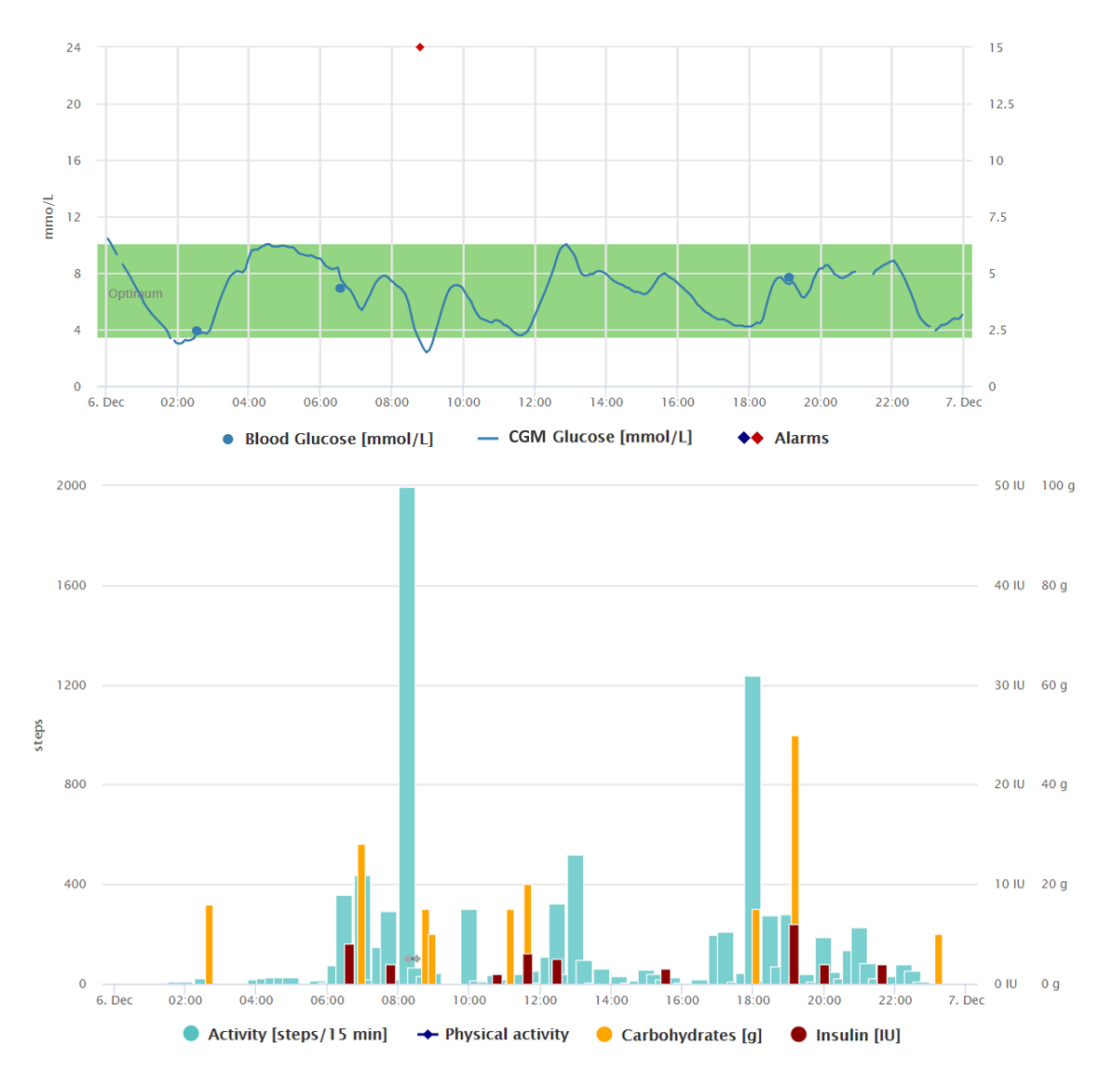
Graphical interpretation of 1-day data registrations of the patient from case study 3, visualized by the Diani Web app. In the picture, we can notice variable activities during the day composed of 1 high-intensity physical activity at around 8 am and then frequent changes between sitting and walking patterns. Regarding food intake, there are very different amounts of carbohydrates throughout the day. Patient´s reaction on blood glucose level made by correction boluses is another typical pattern. CGM: continuous glucose monitoring.

Being connected fulltime through her CGM device since 2017 and having the motivation to undergo educational courses, she has learned a lot about how to make insulin adjustments based upon the arrow trends and actual glucose readings on her CGM system. Therefore, based on the trends, she gives herself correction boluses or suspends the basal rate often, rather than exactly counting carbohydrates in foods and reacting on upcoming situations too much in advance (see Figure 4).

She is able to register data into the diary properly as far as she has a good reason for doing so (eg, her blood glucose is suddenly, for unknown reasons, out of her control) and has a sufficient motivation (such as being pushed by her diabetes friends).

To keep her blood glucose in a very tight target range, she sets the hypo- and hyperglycemia ranges close to each other. This naturally results in more frequent alarms coming out of the CGM receiver, but it does not disturb her daily activities. However, this becomes a problem during the nights when she often does not hear the alarm and, thus, often does not wake up because of unstable glycemia values. To ensure that she wakes up once the alarm goes off, she started to change the tones in her new receiver settings every week once she got the version enabling that.

Although she is keen on trying new diabetes-related or fitness devices, she often comes up against technical issues, especially with her mobile phone. She uses many social media apps and games, listens to music, and uses a navigation app when driving a car, so she always needs an extra secure digital card and a phone that enables her to always be *online*. Lack of signal to connected devices is also a frequent issue she faces. Another issue is the fact that she lost some devices in the past, which were too tiny or not well fixed to her body, or the devices simply stopped working because of unknown reasons. Besides that, she is able to figure out most of the technical issues she encounters.

##### Suggestions for the Optimal Combination of Devices

Knowing all this information, this patient would certainly benefit from CGM technology and rely on its real-time values. Insulin pump is also the most convenient tool for her. However, as we know, losing the Bluetooth signal on her phone is a frequent issue. Therefore, she might benefit a lot from a pump that displays blood glucose values directly on the pump. An even better option would be a combination of displaying the data both on the pump and on the phone (similar to the previous case no. 2), as that would ensure the ability to arbitrarily change the alarm tone on her phone, in addition to receiving the 1-tone alarm from the pump. In connection to her ability to do her correction boluses based upon the glucose values and trends displayed on the pump, she could benefit from using a device that would automatically suspend the dosing before her glucose is predicted to reach a low level. It could help her to reduce hypoglycemia incidents (see Table 2), especially when overdone correction boluses occur. Considering a hybrid closed-loop system, if available in patient’s country, this patient might have trouble handling its automatic delivery function because she might tend to manually interrupt its action mostly on high glucose levels to assume control of the insulin corrections and make the action faster.

#### Case Study 4

##### Patient Information

A 27-year-old man has T1DM since 1992, and he has been on CSII regimen since 2002. He started using the Diani system in July 2014, starting with the HbA_1c_ of 78 mmol/mol. Other than a laser operation he underwent because of early manifestations of retinopathy, he is not suffering from other diseases or diabetes complications.

##### Daily Regimen and Self-Management

Working in an administration office, this patient has a mostly regular working schedule. In his free time, he is physically very active (performing regular PA, such as floorball and volleyball). He is also very creative and likes gaming. He is a very competitive person when it comes to any games or sport matches.

Regarding his eating habits, he often underestimates the timing of insulin injection for meals and sometime takes boluses too late. He also loves beer and is on a bit higher carbohydrate diet, which causes frequent postmeal spikes.

He tries to check his blood glucose regularly, but there are some days with only 1 or no measurements.

##### Patient’s Attitude to Technology

This patient gets the sensor for CGM few times a year, but most of the time, he relies on SMBG alone. He is very competent technically and is able to learn, even intuitively, how to operate new mobile apps or wearables. He is also able to solve common problems with loss of connection and other minor technical complications on his own.

Considering his competitiveness, he is using an activity tracker all the time to set goals and compete with his friends in daily/weekly step counts.

However, it is difficult for him to register data into the diary manually. He is willing to enter more data when he gets motivation from the outside that, in addition, is often updated by some new stimulus. It can be a new tailored version of the app or another app that has a game basis, but it can also be a new device itself (a new *toy* he can play with). With respect to the long-term motivation, the best chance for him to better self-manage his disease is competing with his diabetic friends.

**Figure 5:**
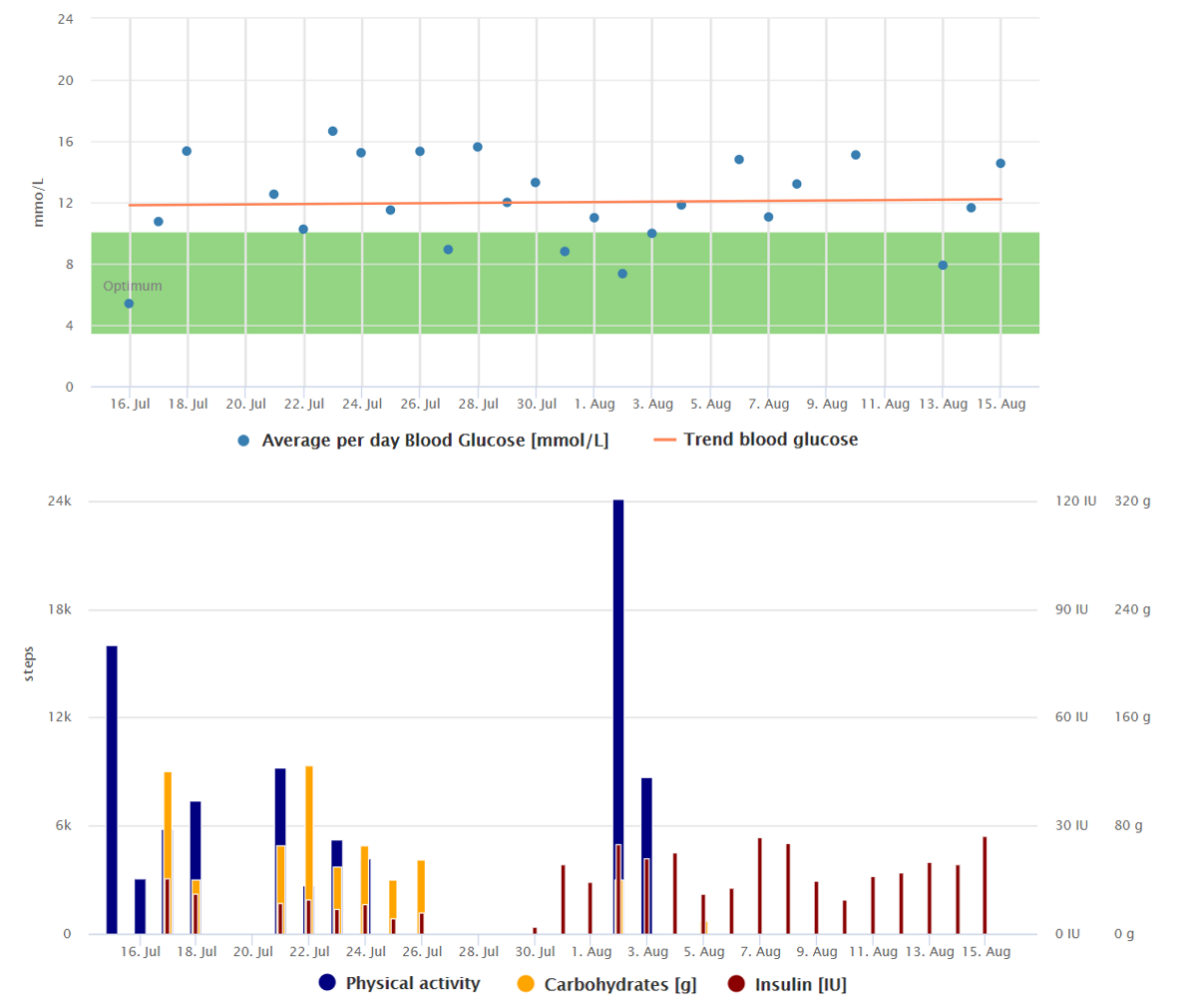
Graphical interpretation of 1-month data registrations of the patient from the case study 4, visualized by the Diani Web app. In the picture, we can observe irregular data registrations and high blood glucose variability.

**Figure 6:**
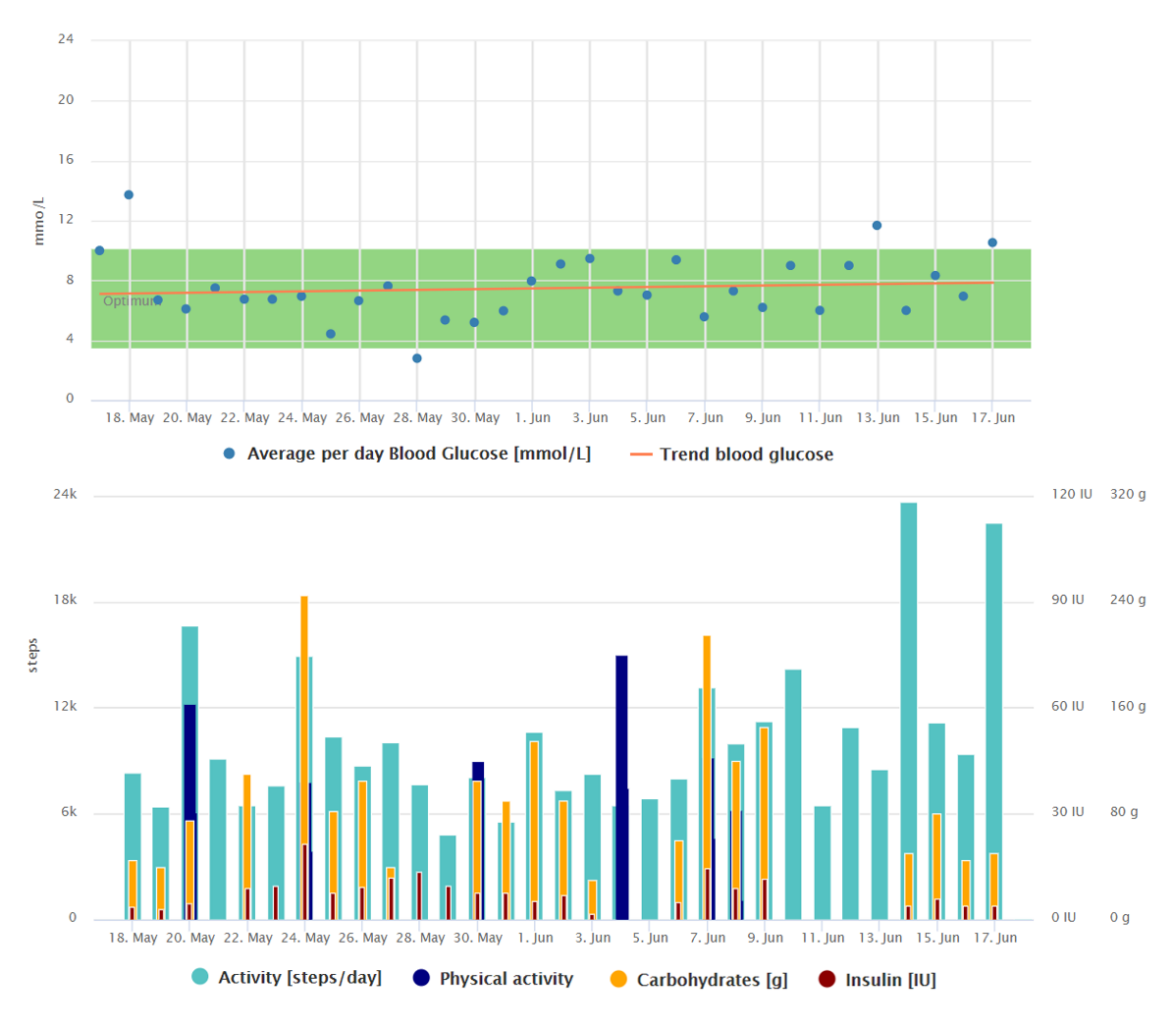
Graphical interpretation of 1-month data registrations of the patient from case study 4 after the patient got the activity tracker. The frequency of data registrations has increased, and the average blood glucose has decreased significantly compared with the average blood glucose trend in Figure 5.

Figures 5 and 6 show the difference between the phase when he was not wearing the activity tracker and the period after he got the new device.

Although he uses just basic functions of his insulin pump, he has no problem to operate any kind of device and could benefit from more advanced functions (eg, square wave or dual wave bolus, bolus wizard) in case he got proper education in diabetes management.

##### Suggestions for the Optimal Combination of Devices

Considering all these aspects, patients similar to this one might try to use, for example, a mobile app that is gamesome, includes functions for setting challenges, and enables users to take advantage of coaching services through a certified diabetes educator to increase the level of education in self-management issues. This patient might also be a good candidate for a hybrid closed-loop system, as it could reduce the burden of frequent blood glucose control and reduce the postmeal spikes caused by his eating habits and incorrect bolusing time.

If the hybrid closed-loop system is not accessible, we could consider a pump that suspends before low (it means the insulin delivery is stopped when low blood glucose limit is predicted to be reached within certain time). This could, in addition to other benefits, reduce hypoglycemia incidents, especially during PA. However, if the chosen pump is susceptible to falls and hits, the silicon case should be in place to protect the device while performing competitive sports.

#### Case Study 5

##### Patient Information

A 45-year-old man was diagnosed with T1DM in 2014. He has been on an MDI regimen right from the onset of the disease. Apart from diabetes and arterial hypertension, he is not suffering from other diseases or diabetes complications.

##### Daily Regimen and Self-Management

The patient has an irregular working regimen because of his frequent nighttime shifts. However, he performs PAs (walking, cycling, and working out) regularly and more than twice a week. He is used to complying with a fixed daily insulin dosing and tries to do proper carbohydrate counting using the nutrition tables on food packages.

##### Patient’s Attitude to Technology

Before starting to use the Diani system, this patient had never used any mobile or Web app for diabetes self-management. Therefore, the only data he could check were the values displayed on his glucometer, which he had never reviewed before.

However, he had no problem with handling the smartphone and learning how to work with the mobile app and make data registration. He only had some minor issues when connecting the smartwatch to his phone at the very beginning, which he was able to manage on its own quickly once he got the proper instructions for the device pairing process.

**Figure 7:**
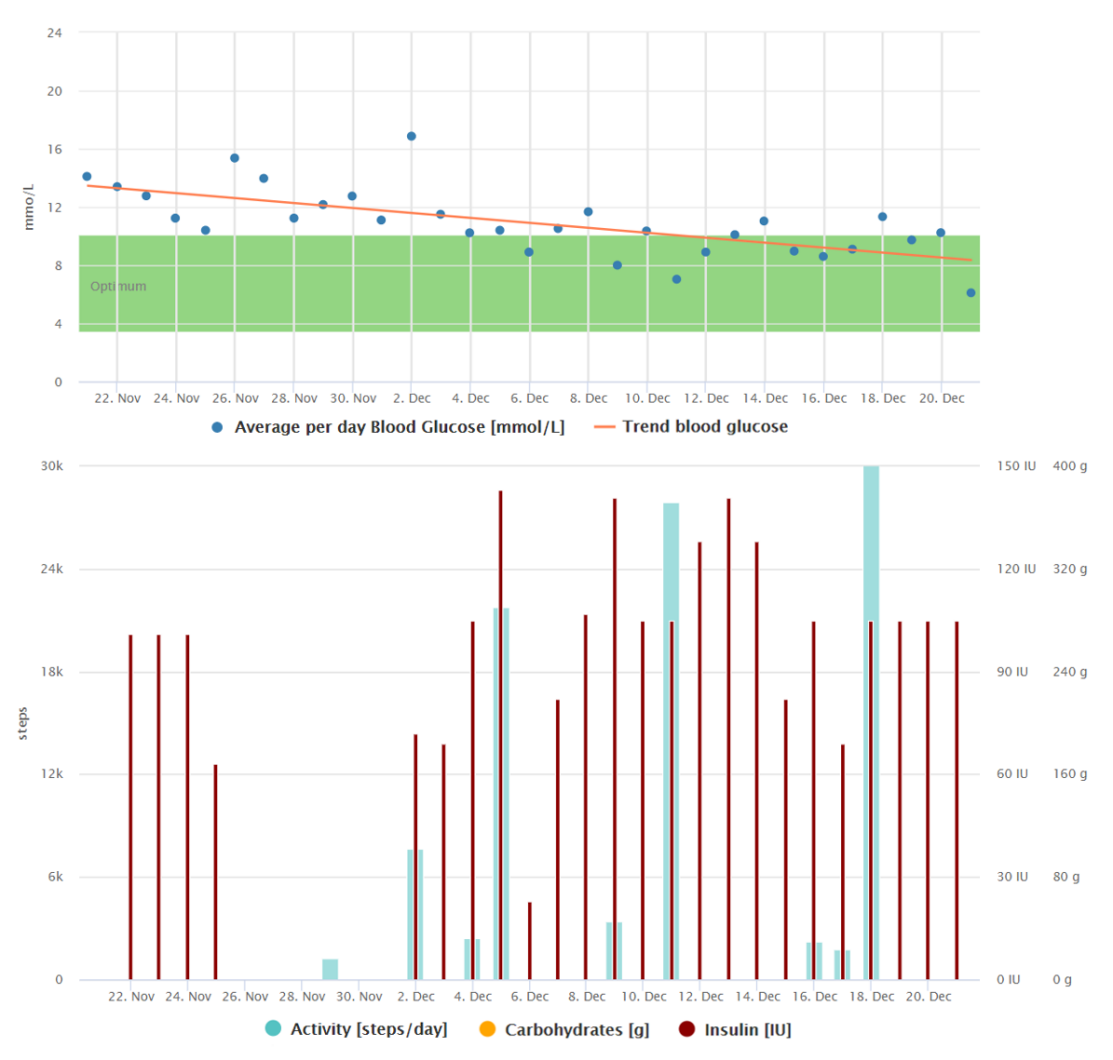
Graphical interpretation of 1-month data registrations of the patient from case study 5, visualized by the Diani Web app. We can observe the decreasing trend of average daily blood glucose during the first month of using the telemedicine system.

**Figure 8:**
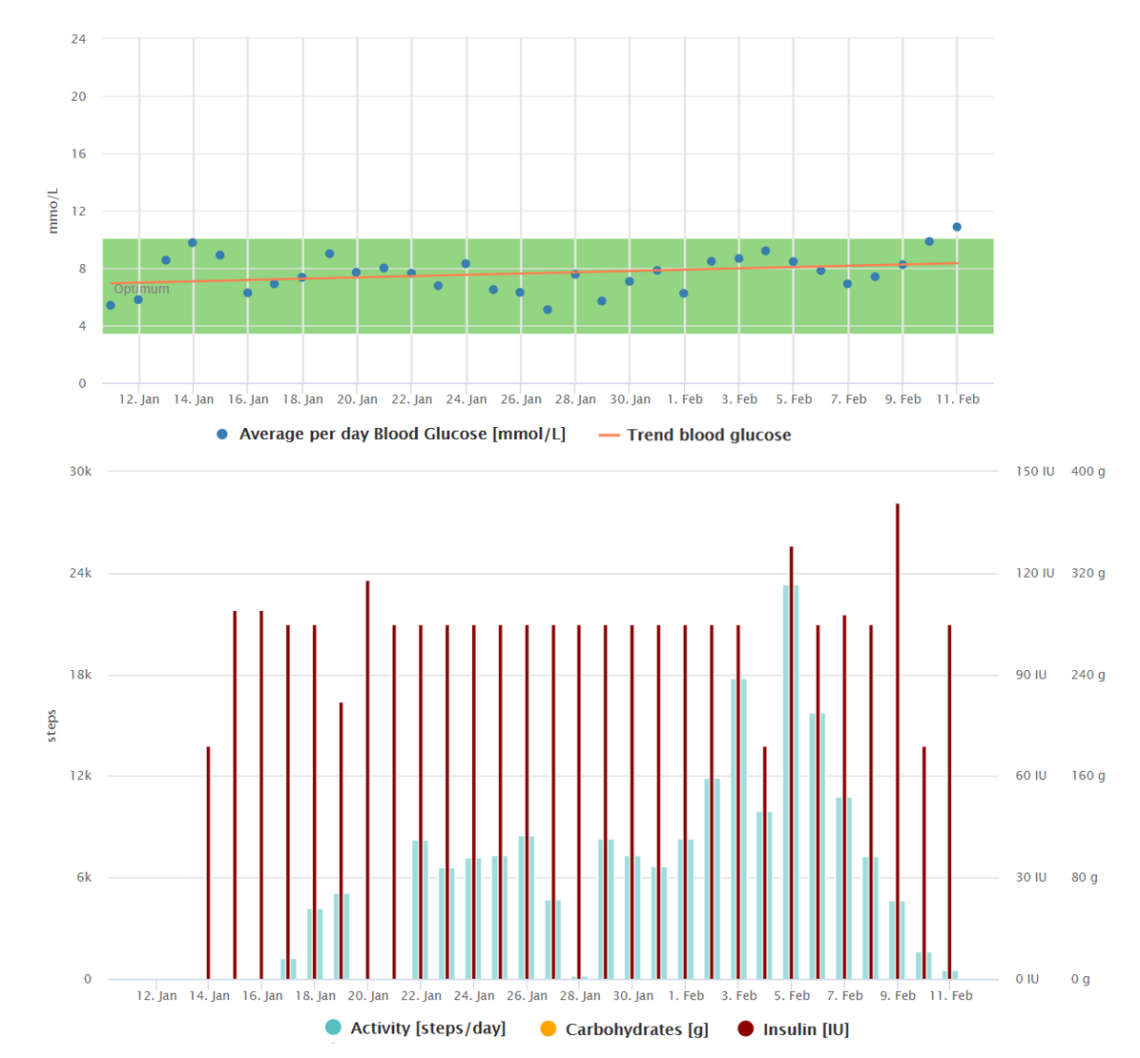
Graphical interpretation of 1-month data registrations of the patient from case study 5 after 2.5 months of using the telemedicine system. We can see the data registrations are more regular than those in Figure 7, and the average blood glucose has decreased and stabilized.

The diabetes diary mobile app was the most beneficial tool for him as it was very easy to operate, the data were displayed in a well-arranged way, and the app was in his native language. Reviewing his data, he was motivated to achieve better results (changes in average blood glucose trends can be observed when comparing Figures 7 and 8). He was very happy about the automatic transfer of data from the glucometer to his phone. Insulin doses were, with the exception of a few PA comments, the only data manually registered by him (see Figures 7 and 8). On the basis of the last consultation, this patient feels much better both physically and mentally, and his quality of life has improved appreciably. He would also be willing to pay a monthly fee for the telemedicine service.

##### Suggestions for the Optimal Combination of Devices

As we can see, the patient was satisfied with the set of devices he was equipped with when using the telemedicine system. As an improvement, we could suggest to him to use an insulin pen that enables automatic transfer of data to the same mobile app into which the data from his glucometer are sent. Potentially, we could discuss how acceptable it would be for him to switch to an insulin pump that would be connected to a blood glucose meter and transfer the measured data to a user-friendly mobile app. The app should also have a bolus calculator and connected food database included to help him to better manage the carbohydrate counting.

#### Case Study 6

##### Patient Information

An 87-year-old man was diagnosed with T1DM in 1982, and he is still on an MDI regimen and SMBG measurement only. He suffers from diabetic peripheral polyneuropathy because of which his hands shake slightly, and he complains of leg pains, for which he takes the prescribed pills. He also suffers from diabetic retinopathy, proteinuria, corrected arterial hypertension, and hypercholesterolemia.

##### Daily Regimen and Self-Management

This patient is retired and as such has a very regular daily regimen and lots of free time he can spend on his hobbies and on the diabetes self-management. He is extremely motivated to learn new things and still has lots of energy to try or read about new methods in diabetes care. Thanks to his caring wife and his own carefulness and sense of precision, he has been keeping his paper-based diabetes records since the onset of his disease. He has read most of the diabetes books available in his native language and made notes about any unusual information that could help him to better self-manage the disease. He also performs daily PA (everyday walking, gardening, and working in his workshop). He was not used to checking his blood glucose regularly before he started to use the telemedicine system (1.7 times per day on average, calculated from 3-month records in his glucometer measured before he started to use the telemedicine system).

##### Patient’s Attitude to Technology

He has already tried multiple types of activity trackers in the past. The device motivates him to maintain regular PA (walking or gardening). He is also able to use a personal computer to a certain extent, that is, for surfing on the internet, sending emails, and using Skype.

For him, the biggest problem he faced with the telemedicine system was that he had never used a smartphone before. Therefore, he first got the phone only to learn how to switch it on and off and how to open the diabetes diary app and use it. After a month, he came back to get the rest of the devices. As he only had a cable internet connection at home, he also got a subscriber identity module (SIM) card with prepaid data with the phone. Despite his efforts to handle the phone, he often had problems with operating, charging the devices, and losing the internet connection.

**Figure 9:**
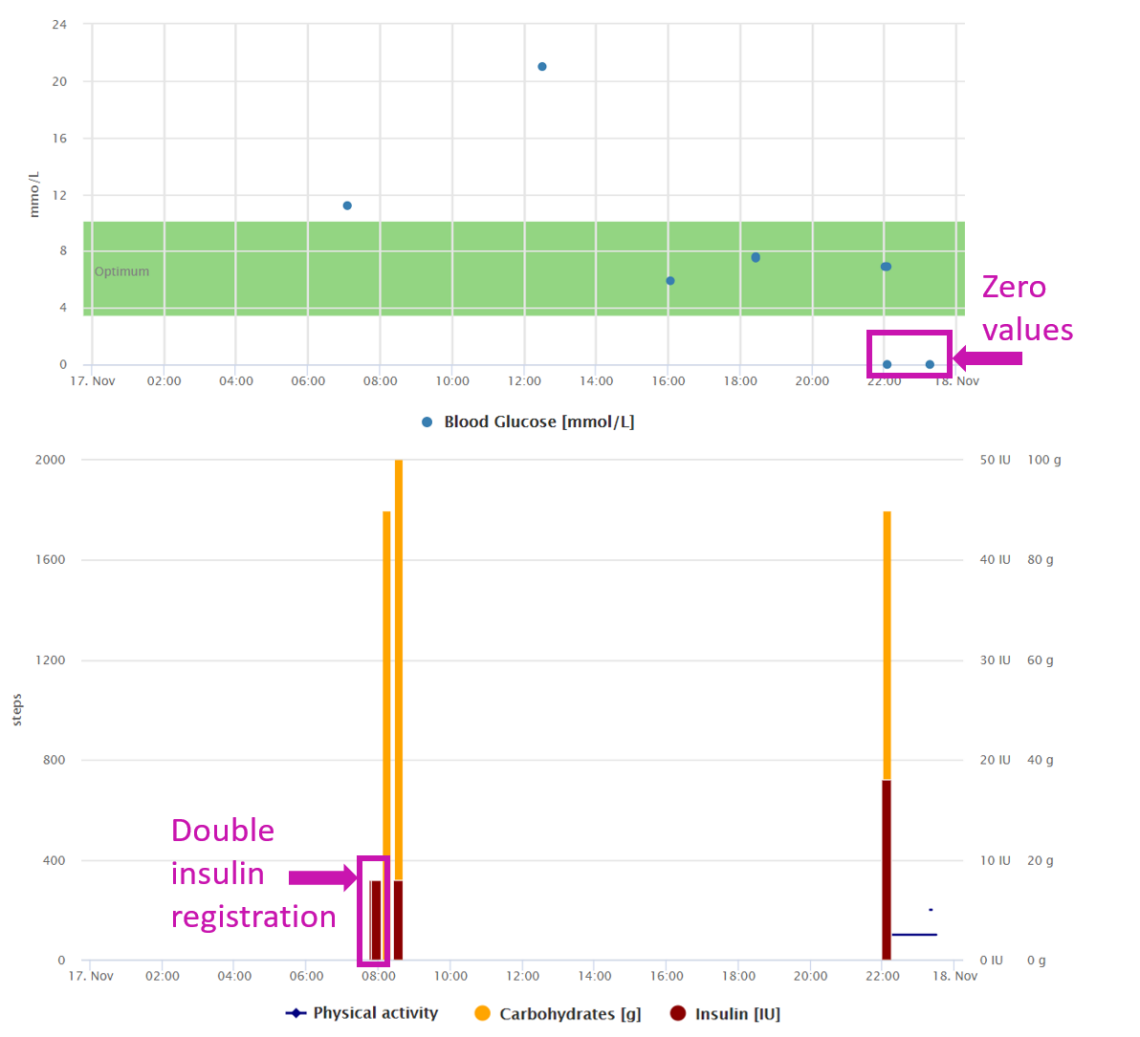
Graphical interpretation of 1-day data registrations of the patient from case study 6. We can see some accidentally duplicated or zero values because of the patient’s inability to operate the mobile app.

As he suffered from neuropathy, it was also difficult for him to handle the touchscreen because his hands were shaking, and he often clicked on more than 1 button at the same time or on the wrong one. This led to a wrong data entry (see Figure 9) or frequent calls to the technical support when getting to a page he did not know how to get out of. Thus, he spent more time dealing with technical issues than using the system effectively.

##### Suggestion for the Optimal Combination of Devices

In summary, besides the activity tracker that is simple to use even for an elderly person, the rest of the system is not a suitable solution for such a patient.

Another possible help could be occasional phone call checkups made by some diabetes educator to increase his adherence to regularly checking his blood glucose or connection to a remote assistance service that would track and control the patient via a smart device (SIM card and global system for mobile communication module based) and react in case of emergency.

## Discussion

### Principal Findings

It is obvious, based on the case studies presented here, that there is no *one-size-fits-all* diabetes self-management tool, which would fully satisfy the needs of each particular patient.

To be able to give proper advice about the type of device that would be the most suitable for a given patient, specific information about the person is required. Such information includes, in particular, the patient’s personality, his/her technical skills, daily regimen, his/her attitude to diabetes, obstacles in diabetes management, preferences in data visualization and devices’ functionalities, willingness to learn new things, and motivational means that could help him/her use any system effectively and on a long-term basis.

From the case series, we could learn that a physically very active patient, who tends to conceal the disease from public, could only benefit from a technology that would not represent an obstacle when performing PA and would not be visible from the outside. On the contrary, there are patients who do not have any problems wearing any kind of devices and of any size, as far as the system is reliable and sufficiently accurate.

Although some patients would benefit from automatic functions of the most advanced pumps that suspend the insulin delivery, or work in hybrid mode, enabling them to reduce their hypoglycemia incidents and correcting their *mistakes* in bolusing, there are other patients for whom this system could be rather burdensome. These are patients who need to have their dosing under control, do not trust the system, and do not have the will to wait until the system corrects their blood glucose spikes.

Technical abilities, educational level, and age can also play a dominant role in technology acceptance and its use. Obviously, there are certain limits indicating that a given patient would not be able to operate some systems, even if proper education and technical support were provided.

Motivational tool embedded into technology is more related to a patient’s personality. It can be, for example, the ability to share data with other patients/users or compete with friends who constantly push a patient to achieve good results, a function that is gamesome, or even just the ability to review data from the given device that proves to the patient that he or she is doing well or has achieved exceptional results.

It is also very effective to observe how patients handle a given technology in the present and after they get a new one and to check the data they collect regularly to learn more about their daily regimen, self-management, and potential changes in data entry and frequency of data registration over the long term.

With respect to the effect of the telemedicine system on patients’ self-management and blood glucose value improvements, we can see a reduction in HbA_1c_ values in all but 1 of the 6 patients. However, because the patients were using the system in different periods of time, and because there could also be other factors that were not tracked but could have an impact on HbA_1c_ reduction, we cannot attribute this effect to our system only.

### Conclusions

We believe this paper can provide relevant guidance on how to help particular patients to choose the best technology that is likely to suit them the most based on specific patient information we are able to obtain.

The input information we get about a patient represents one of the sources clinicians or educators can use to help a given patient to find the optimal combination of devices for diabetes self-management. Furthermore, clinicians or educators should be aware of available technologies a given patient can choose from. In addition, to achieve an effective use of chosen devices, there is a substantial need for proper education of the patients before they start using them.

Furthermore, with the rapid development of new and more advanced technological solutions (ie, hybrid and closed-loop systems), educators specialized in technical-related areas of diabetes management are needed to help with such customization and technical support of patients.

## Appendix

Multimedia Appendix 1 Screenshots of the Diani Web application.

## Abbreviations

CGM: continuous glucose monitor
CSII: continuous subcutaneous insulin infusion
HbA_1c_: glycated hemoglobin
MDI: multiple daily injection
PA: physical activity
SIM: subscriber identity module
SMBG: self-measured blood glucose
T1DM: type 1 diabetes mellitus
