# Supplementary material for: Customizing the Types of Technologies Used by Patients With Type 1 Diabetes Mellitus for Diabetes Treatment: Case Series on Patient Experience
Source: JMIR Mhealth Uhealth. 2019 Jul 9;7(7):e11527. doi: 10.2196/11527 (PMC6647757; doi:10.2196/11527)
Supplement: Multimedia Appendix 1 [file mhealth_v7i7e11527_app1.pdf]

Diani web portal

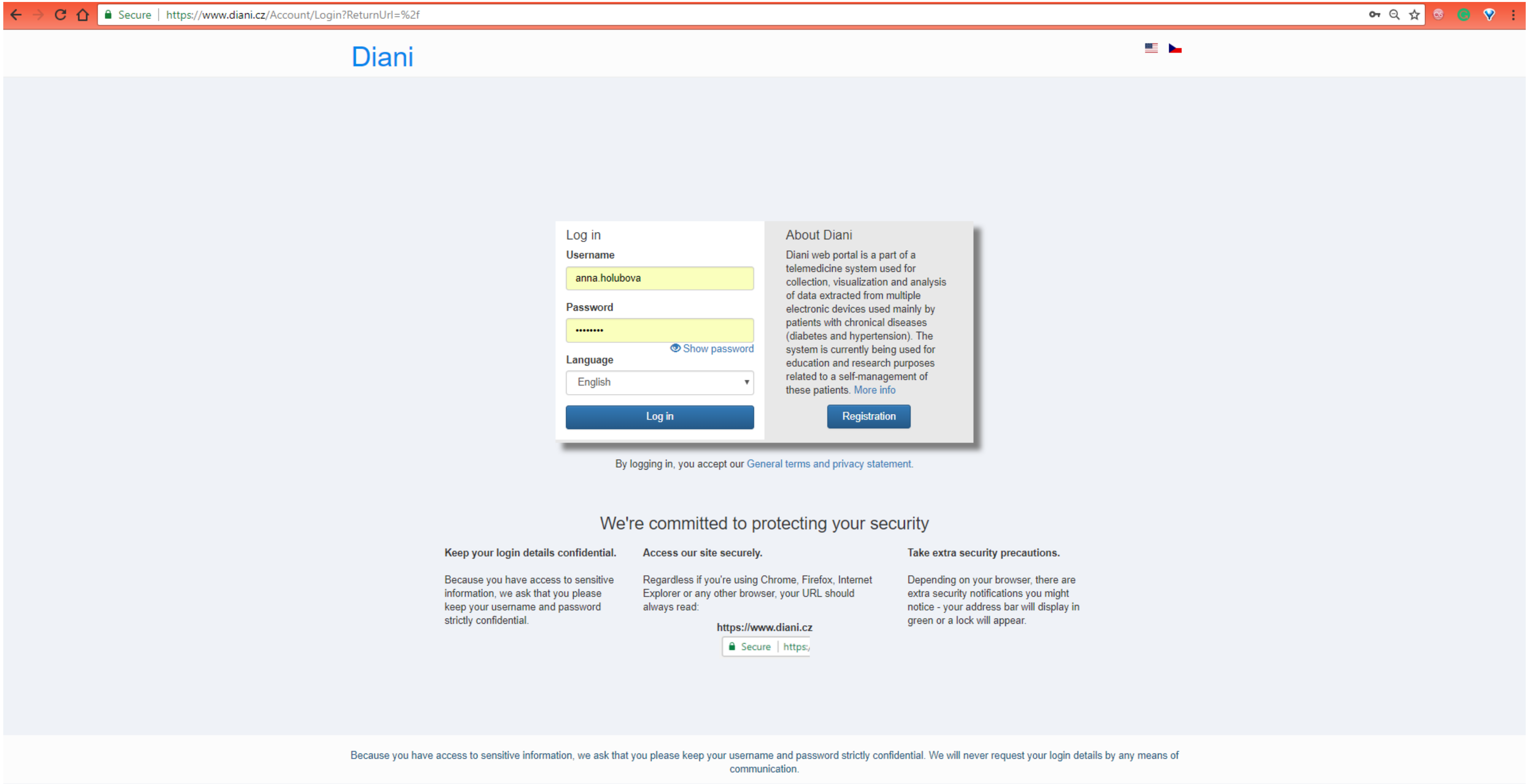

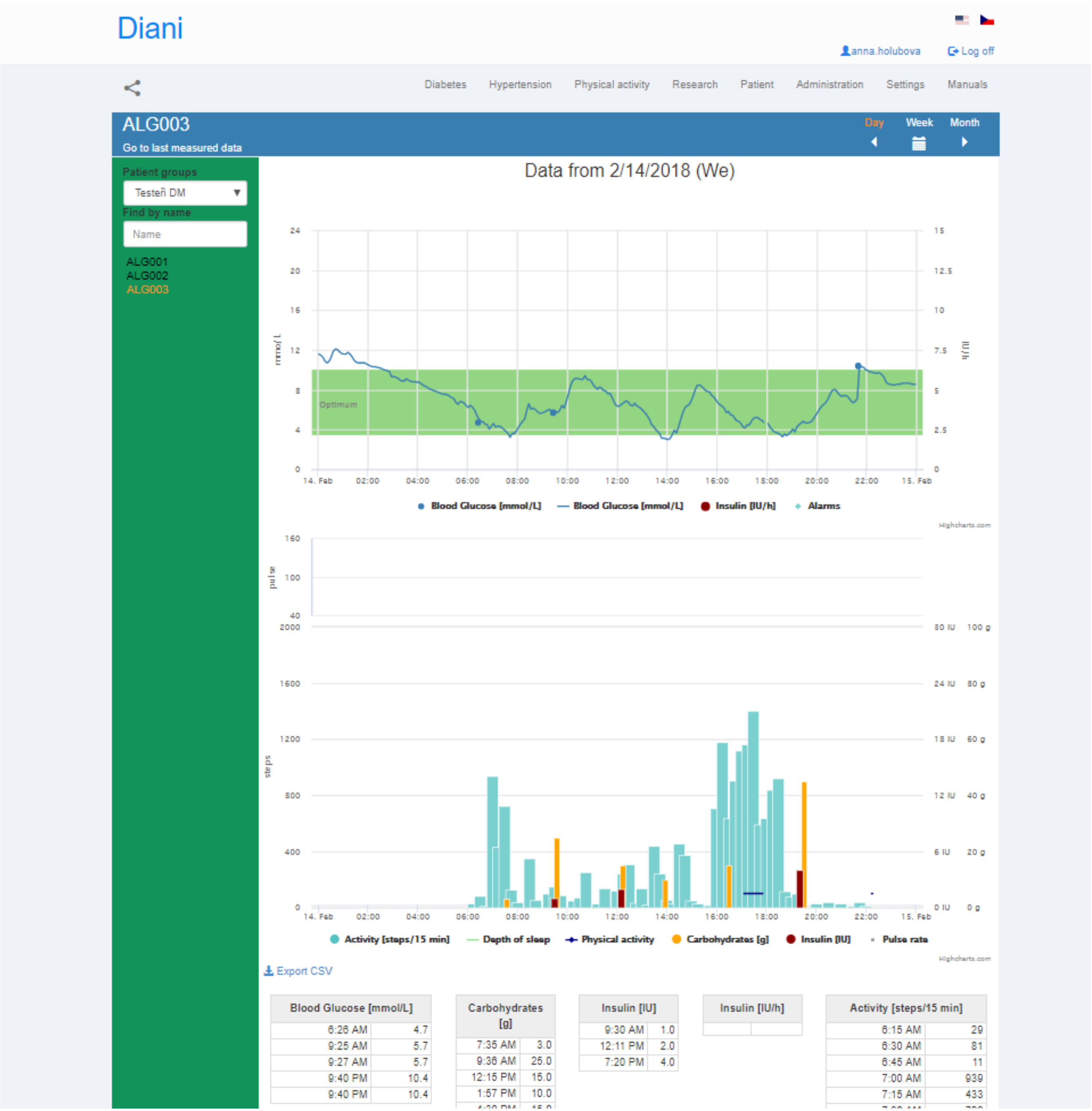
Diani
anna.holubova
Log off
Diabetes
Hypertension
Physical activity
Research
Patient
Administration
Settings
Manuals
ALG003
Go to last measured data
Day
Week
Month
Patient groups
Testeři DM
Find by name
Name
ALG001
ALG002
ALG003
Data from 2/14/2018 (We)
Optimum
Blood Glucose [mmol/L]
Blood Glucose [mmol/L]
Insulin [IU/h]
Alarms
Activity [steps/15 min]
Depth of sleep
Physical activity
Carbohydrates [g]
Insulin [IU]
Pulse rate
Export CSV
Blood Glucose [mmol/L]
Carbohydrates [g]
Insulin [IU]
Insulin [IU/h]
Activity [steps/15 min]

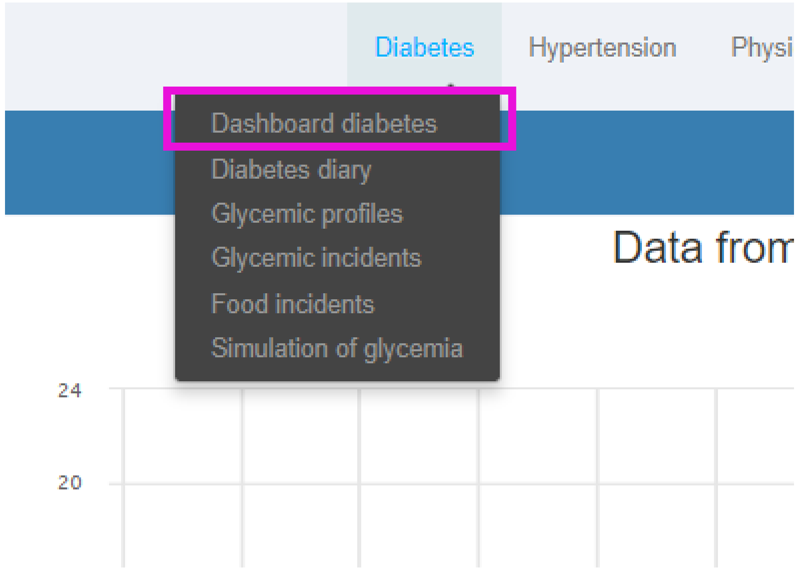
Diabetes
Hypertension
Physi
Dashboard diabetes
Diabetes diary
Glycemic profiles
Glycemic incidents
Food incidents
Simulation of glycemia
Data from
24
20

Diani

anna.holubova Log off

Diabetes Hypertension Physical activity Research Patient Administration Settings Manuals

Diabetes diary

Selected client: ALG003 From: 02/14/2018 To: 02/18/2018 Days: 5 Generate Export to Pdf

								Breakfast					Lunch					Dinner								
**Date**	**Type**	**0**	**1**	**2**	**3**	**4**	**5**	**6**	**7**	**8**	**9**	**10**	**11**	**12**	**13**	**14**	**15**	**16**	**17**	**18**	**19**	**20**	**21**	**22**	**23**	**Note**
Wednesday 2/14/2018	BG							4.7			5.7												10.4			
	CAR								3		25			15	10			15			45					
	INS										1.0			2.0							4.0					
	ACT																		Zumba*							
	STEPS								2226									3426	4279	2508						
Thursday 2/15/2018	BG							7.9			4.8					5.5				3.8	5.0					
	CAR								3			10		6				40					45			
	INS							1.0					2.0								4.0					
	ACT																		Funkcni trenink**	Latinos*						
	STEPS								2692							2410	1046	2533	1844	3667	1162					
Friday 2/16/2018	BG							3.3													6.2					
	CAR								20	3			3	10	0			20			55					
	INS													1.0							5.0					
	ACT																			Body*						
	STEPS								1249	1475					1961	4188	2539	1679	2420	2223						
Saturday 2/17/2018	BG								6.5	6.5			4.7							6.7						
	CAR										15		20	15				7			40	10				
	INS									1.0				2.0						1.0	4.0					
	ACT												Intervalovy trenink**													
	STEPS										1722	2076	2036				1154	1130								
Sunday 2/18/2018	BG																									
	CAR																									
	INS																									
	ACT																									
	STEPS										1627	2571	4317	1205		3547			2520	1975						

Date: 7/9/2018 Time: 20:40:44 Baterry: 14% Signal strength: -

Legend

Low Blood Glucose	<= 4 (mmol/L)	(red)
Normal Blood Glucose	4 - 10 (mmol/L)	(green)
Elevated Blood Glucose	10 - 15 (mmol/L)	(yellow)
High Blood Glucose	>= 15 (mmol/L)	(orange)
Display steps treshold	1000 (steps/h)	

Diabetes Hypertension Physi

- Dashboard diabetes
- Diabetes diary
- Glycemic profiles
- Glycemic incidents
- Food incidents
- Simulation of glycemia

Data from

24

20

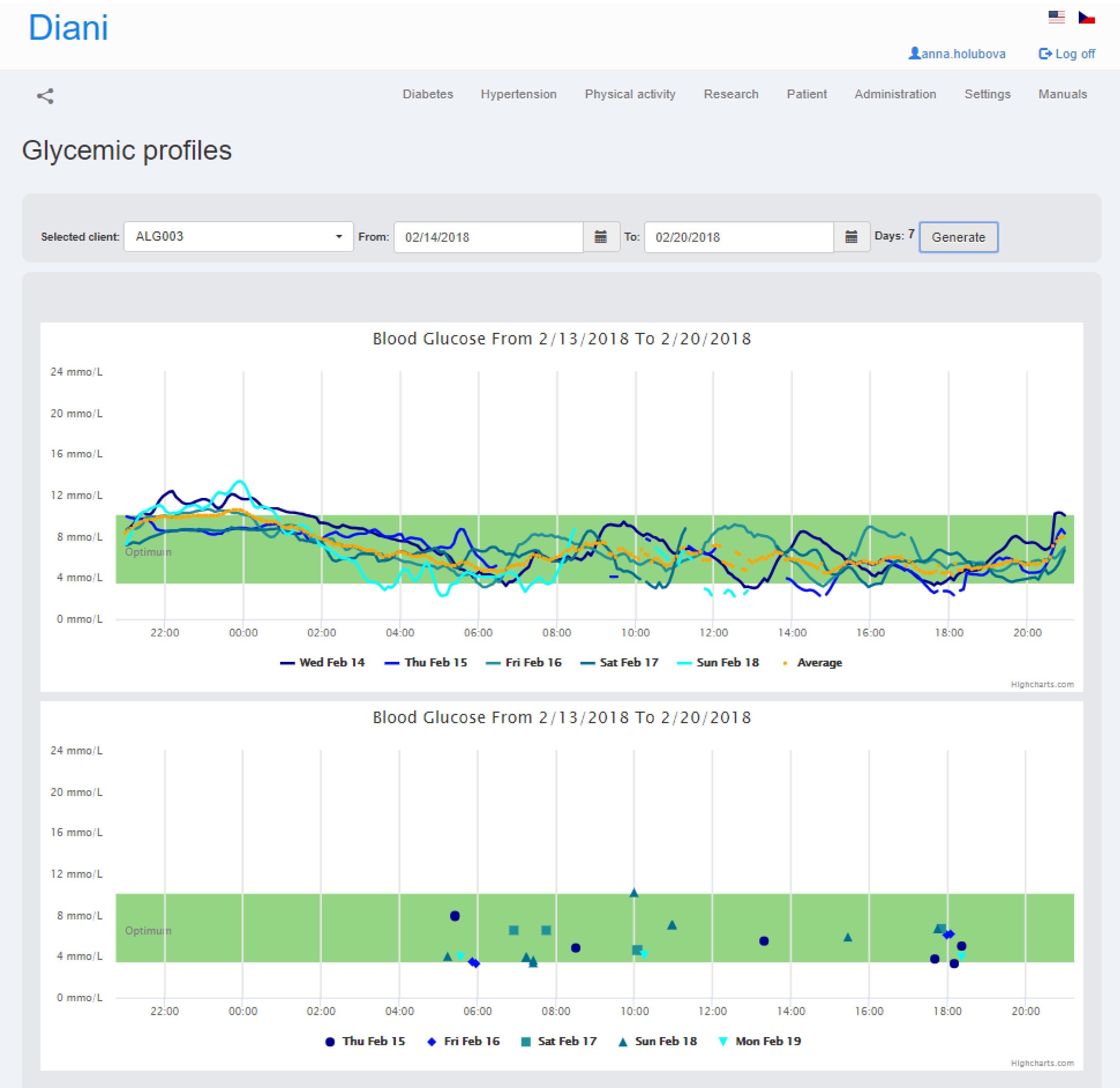
Diani
anna.holubova
Log off
Diabetes
Hypertension
Physical activity
Research
Patient
Administration
Settings
Manuals
Glycemic profiles
Selected client: ALG003
From: 02/14/2018
To: 02/20/2018
Days: 7
Generate
Blood Glucose From 2/13/2018 To 2/20/2018
24 mmo/L
20 mmo/L
16 mmo/L
12 mmo/L
8 mmo/L
4 mmo/L
0 mmo/L
Optimum
22:00
00:00
02:00
04:00
06:00
08:00
10:00
12:00
14:00
16:00
18:00
20:00
Wed Feb 14
Thu Feb 15
Fri Feb 16
Sat Feb 17
Sun Feb 18
Average
Highcharts.com
Blood Glucose From 2/13/2018 To 2/20/2018
24 mmo/L
20 mmo/L
16 mmo/L
12 mmo/L
8 mmo/L
4 mmo/L
0 mmo/L
Optimum
22:00
00:00
02:00
04:00
06:00
08:00
10:00
12:00
14:00
16:00
18:00
20:00
Thu Feb 15
Fri Feb 16
Sat Feb 17
Sun Feb 18
Mon Feb 19
Highcharts.com

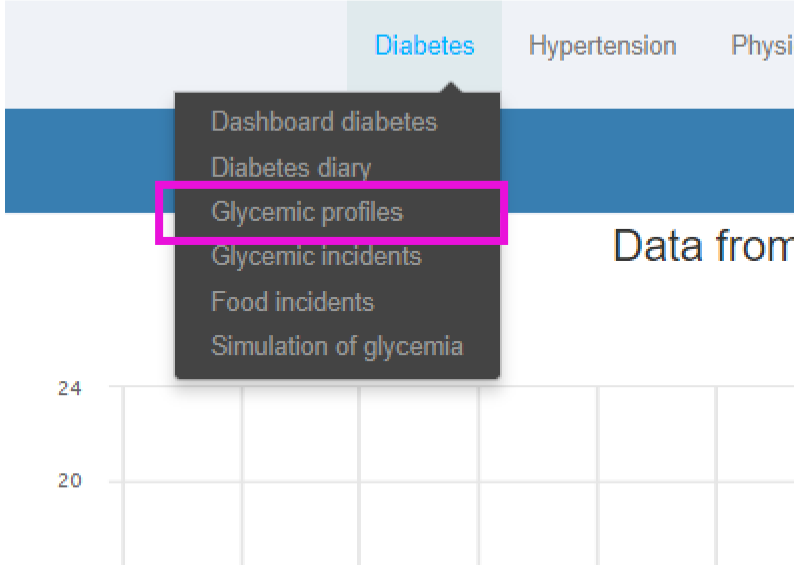
Diabetes
Hypertension
Physi
Dashboard diabetes
Diabetes diary
Glycemic profiles
Glycemic incidents
Food incidents
Simulation of glycemia
Data from
24
20
